# Contamination of hospital surfaces with respiratory pathogens in Bangladesh

**DOI:** 10.1371/journal.pone.0224065

**Published:** 2019-10-28

**Authors:** Md. Zakiul Hassan, Katharine Sturm-Ramirez, Mohammad Ziaur Rahman, Kamal Hossain, Mohammad Abdul Aleem, Mejbah Uddin Bhuiyan, Md. Muzahidul Islam, Mahmudur Rahman, Emily S. Gurley

**Affiliations:** 1 icddr,b (formerly, International Centre for Diarrheal Disease Research, Bangladesh), Dhaka, Bangladesh; 2 School of Public Health and Community Medicine, University of New South Wales, Sydney, NSW, Australia; 3 Division of Paediatrics, School of Medicine, The University of Western Australia, Perth, WA, Australia; 4 Institute of Epidemiology, Disease Control and Research (IEDCR), Dhaka, Bangladesh; 5 Department of Epidemiology, Johns Hopkins Bloomberg School of Public Health, Baltimore, Maryland, United States of America; University Hospital Basel, SWITZERLAND

## Abstract

With limited infection control practices in overcrowded Bangladeshi hospitals, surfaces may play an important role in the transmission of respiratory pathogens in hospital wards and pose a serious risk of infection for patients, health care workers, caregivers and visitors. In this study, we aimed to identify if surfaces near hospitalized patients with respiratory infections were contaminated with respiratory pathogens and to identify which surfaces were most commonly contaminated. Between September-November 2013, we collected respiratory (nasopharyngeal and oropharyngeal) swabs from patients hospitalized with respiratory illness in adult medicine and paediatric medicine wards at two public tertiary care hospitals in Bangladesh. We collected surface swabs from up to five surfaces near each case-patient including: the wall, bed rail, bed sheet, clinical file, and multipurpose towel used for care giving purposes. We tested swabs using real-time multiplex PCR for 19 viral and 12 bacterial pathogens. Case-patients with at least one pathogen detected had corresponding surface swabs tested for those same pathogens. Of 104 patients tested, 79 had a laboratory-confirmed respiratory pathogen. Of the 287 swabs collected from surfaces near these patients, 133 (46%) had evidence of contamination with at least one pathogen. The most commonly contaminated surfaces were the bed sheet and the towel. Sixty-two percent of patients with a laboratory-confirmed respiratory pathgen (49/79) had detectable viral or bacterial nucleic acid on at least one surface. *Klebsiella pneumoniae* was the most frequently detected pathogen on both respiratory swabs (32%, 33/104) and on surfaces near patients positive for this organism (97%, 32/33). Surfaces near patients hospitalized with respiratory infections were frequently contaminated by pathogens, with *Klebsiella pneumoniae* being most common, highlighting the potential for transmission of respiratory pathogens via surfaces. Efforts to introduce routine cleaning in wards may be a feasible strategy to improve infection control, given that severe space constraints prohibit cohorting patients with respiratory illness.

## Introduction

Pathogens present in ill patients' respiratory secretions can contaminate nearby hospital surfaces, such as floors, walls, bedrails and mattresses, through coughing, sneezing and touching [1–4]. Respiratory viral and bacterial pathogens, including *Staphylococcus aureus*, *Streptococcus pyogenes*, influenza viruses, respiratory syncytial virus, adenovirus, rhinoviruses and novel coronavirus strains, can survive on hospital surfaces for days, weeks or even months. Furthermore, touching contaminated surfaces may lead to nosocomial transmission of pathogens between patients, family caregivers, visitors, and healthcare workers [1, 5, 6].

Patient care areas in Bangladeshi hospitals are open wards with multiple beds in a room and are frequently overcrowded with patients, family caregivers, and visitors [7, 8]. A previous study by Rimi et al. found a median of four people per 100 sq. feet of floor space in hospital wards in Bangladesh and observed a median of five uncovered coughs or sneezes per 100 sq feet per hour [7]. Due to shortage of health care workers, family caregivers (family members who provides 24 hour hands on care to sick patient, including bedside nursing and cleaning) are integral part of inpatient care in Bangladeshi public hospitals, contributing crowding of hospital wards [8, 9]. The World Bank estimated that in 2014 only $32 of public funds per capita were spent annually on health infrastructure in Bangladesh. Thus, resources for infection control are severely limited in Bangladeshi hospitals [7, 10], making it difficult to implement international infection control guidelines [11].

The lack of routine infection control practices, including no regular surface cleaning, may increase the transmission of respiratory pathogens via hospital surfaces [3, 5, 6, 8]. Family caregivers, visitors, and hospital staff may acquire respiratory infections either through direct contact with infected patients or via droplets, aerosols or contaminated surfaces. Contaminated hospital surfaces can pose a serious risk of infection for patients, health care workers, caregivers and visitors.

Within this context of scarce resources, describing the magnitude of surface contamination in Bangladeshi hospitals, particularly identifying priority areas for decontamination, could influence infection control policy and practice. Our objective was to assess the frequency with which patients hospitalized for respiratory illnesses in Bangladeshi public hospitals contaminate nearby surfaces, to identify commonly contaminated surfaces, and to determine which pathogens are detected most frequently.

## Materials and methods

### Case identification and sample collection

We conducted the study in two public tertiary care teaching hospitals in Rajshahi and Jessore, Bangladesh between September and November 2013. Rajshahi Medical College Hospital contains approximately 1,200 beds with eight adult medicine wards and four pediatric wards. Jessore Medical College Hospital is a 250-bed hospital with two adult medicine wards and one pediatric ward. Paediatric wards typically admit patients <14 years of age and older patients are admitted to adult medicine wards. The mean bed occupancy proportion in these hospital wards are consistently >100% with patients being treated on the floor and in hallways when beds unavailable [8, 12]. Study physicians in adult medicine and pediatric wards identified patients aged ≥5 years who met the severe acute respiratory illness (SARI) case definition of subjective or measured fever (≥38 C°) within the past seven days with cough or sore throat [13]. In pediatric wards, physicians identified children <5 years of age who met the severe pneumonia (SP) case definition: cough or difficulty breathing and at least one danger sign (i.e. chest indrawing, stridor while calm, history of convulsions, inability to drink, lethargy or unconsciousness and/or intractable vomiting) with onset of symptoms within the last seven days [13]. Since case-patients were identified immediately after admission, their illnesses were mostly community acquired.

Study physicians collected respiratory swabs (nasopharyngeal and oropharyngeal) from identified cases using the World Health Organization's laboratory safety manual protocol [14] and pooled them into a single cryovial containing viral transport media (VTM). Trained research assistants in each hospital collected one swab sample from five different surfaces near each enrolled case patient: the wall next to the patient’s bed, bed rail, bed sheet, clinical record files, and a multipurpose towel. The multipurpose towel is a cloth brought from home by family caregivers and used to clean patient respiratory secretions, wiping the patient’s face or head, and to dry caregivers’ hands and face [8]. We selected these surfaces because patients, caregivers, and healthcare workers (HCWs) frequently come into contact with them in the hospital wards [6, 7, 15]. The research assistants collected surface swabs between 12–72 hours after the case-patients’ admission to the hospital. This allowed for adequate time for hospital surfaces to be exposed to possible contamination by respiratory pathogens, while also making sure that surfaces were swabbed before the enrolled patients were discharged or died, as patient turnover in wards was high with a median hospital stay of three days [16]. The risk for infection from these potentially contaminated surfaces between patients within a room, between patients and healthcare workers, or between patients and family caregivers would vary based on the particular surface and how these different risk groups interacted with the surface. With one sterile rayon swab stick per surface, the research assistant swabbed the area of the wall in contact with the bed 45 cm high from the level of the bed sheet, all surfaces of the bed rail located in the area near the patients’ head, half of the bed sheet where the patient’s head was including underneath the patient, front and back cover of the patient file and both sides of the multi-purpose towel. Not all patients had a wall or bedrail nearby as some patients were cared for on the floor, due to overcrowding. Swab samples from each surface area were put into individual cryovials containing VTM and kept in a cool box for up to 30 minutes with a temperature between 2°-8°C. Both the respiratory swabs and surface swab samples were labelled, packaged, stored in a nitrogen dry shipper (-150°C) and sent to the icddr,b virology laboratory by batch twice a month.

### Testing of respiratory and surface swabs

The swab samples were thawed and the cryovials containing the sample were vortexed. About 200 μL of the swab supernatant was used for nucleic acid extraction using InviMag® Pathogen Kit/KF 96 (STRATEC Molecular, Berlin, Germany) and the final eluted volume of nucleic acid solution was 200 μL, as per the manufacturer’s instruction [17]. The real-time multiplex PCR assay was performed as per the manufacturer’s instructions using an AgPath-ID ^™^ One-Step RT-PCR kit (Ambion) with the Fast Track Diagnostic (FTD) respiratory pathogens 33 kit (Fast Track Diagnostics, Luxembourg) for 19 different viruses and 12 bacterial pathogens [18]. Case-patients with at least one pathogen detected in their respiratory swab had corresponding surface swabs tested for those same pathogens. Detection of nucleic acid of at least one similar pathogen on respiratory swab and a nearby surface was defined as contamination of that surface. To investigate wider hospital contamination, we also tested the surface swabs collected near case-patients with no pathogens detected in their respiratory swabs for the most commonly detected pathogens identified on surfaces near patients with detected respiratory pathogens.

### Statistical analysis

We summarized the data using descriptive statistics. We assessed the difference in proportion of pathogen detection in respiratory swabs and surface swabs between adult and paediatric ward patients using Chi-Square test considering Fisher exact test where appropriate. Any association with a p value <0.05 was considered statistically significant.

### Ethical consideration

Study participants (aged ≥18 years) or their legal guardians (if aged <18 years) provided informed written consent. The institutional review board of icddr,b reviewed and approved the study protocol. The Institutional Review Board at the Centers for Disease Control and Prevention (Atlanta, GA, USA) deferred to icddr,b's approval.

## Results

We collected and tested respiratory swabs from 104 patients hospitalized with respiratory illness: 50 SARI cases from adult medicine wards and 54 severe pneumonia cases from paediatric medicine wards. The median age of patients in the adult wards was 32 years (IQR 25–48) and in paediatric wards three months (IQR 2–6). The male-to-female ratio was 2.6:1 (Table 1). Of the 104 patients, 79 (75%) had detectable viral and/or bacterial nucleic acid in their respiratory swabs. Paediatric patients more frequently had one or more detectable pathogen in their respiratory swabs than adult patients (91% versus 60%, p = 0.001). Bacterial pathogens were identified in 76% of adult respiratory swabs. *Klebsiella pneumoniae*, *Streptococcus pneumoniae and Staphylococcus aureus* were most commonly detected during the study period. In contrast, viral pathogens were commonly detected among paediatric patients, including human cytomegalovirus, respiratory syncytial viruses, and human rhinoviruses (Table 1). Clinical features, mean duration of symptom onset to sample collection (3.2 days Vs 3 days) did not vary between patients with a detectable viral and a detectable bacterial nucleic acid in respiratory swabs. Two patients, one with *Klebsiella pneumoniae* and one with *Streptococcus pneumoniae* detected in their respiratory swabs, had been hospitalized at other facilities within two weeks prior to admission to the study hospital, suggesting that these organisms could have been hospital-acquired. Both these patients had abnormal chest X-rays and were diagnosed with severe pneumonia in the study hospital.

**Table 1:** Demographic characteristics of study patients and proportion of patients with a detectable nucleic acid for a specific pathogen in respiratory swabs in two tertiary care hospitals in Bangladesh, September-November, 2013.

Characteristics	Adult ward,	Paediatric ward,
No. of patients, n	50	54
Age, median (IQR)	32 (25–48) years	3(2–6) months
Male, n (%)	41 (82)	34(63)
**Patients with detectable nucleic acid for a specific pathogen in respiratory swab, n (%)**	30 (60)	49 (91)
**Bacterial pathogen, n (%)**		
*Klebsiella pneumoniae*	18 (36)	15(28)
*Streptococcus pneumoniae*	9(18)	14(2.6)
*Staphylococcus aureus*	7(14)	6(11)
*Moraxella catarrhalis*	1(2)	8(15)
*Other bacteria*^*a*^	3(6)	8(16)
**Viral pathogens, n (%)**		
*Human cytomegalovirus*	0(0)	21(39)
*Respiratory syncytial viruses A & B*	0(0)	18(33)
*Human rhinoviruses*	4(8)	10(18)
*Human metapneumoviruses A and B*	2(4)	5(9)
*Human parainfluenza viruses*	2(4)	3(5.5)
*Human coronaviruses*	4(8)	2(3.7)
*Other viruses*^*b*^	3(6)	6(11)
**Patients with any detectable nucleic acid on at least one nearby surface (for adult ward, N = 30, paediatric ward, N = 49), n (%)**	18 (60)	31 (63)

^a^ other bacteria: *Haemophilus influenza* (including type B), *Pneumocystis jiroveci* (fungi), *Bordetella pertussis*

^b^ other viruses: human bocavirus, enterovirus/human parechoviruses, adenovirus and influenza viruses

**Fig 1:**
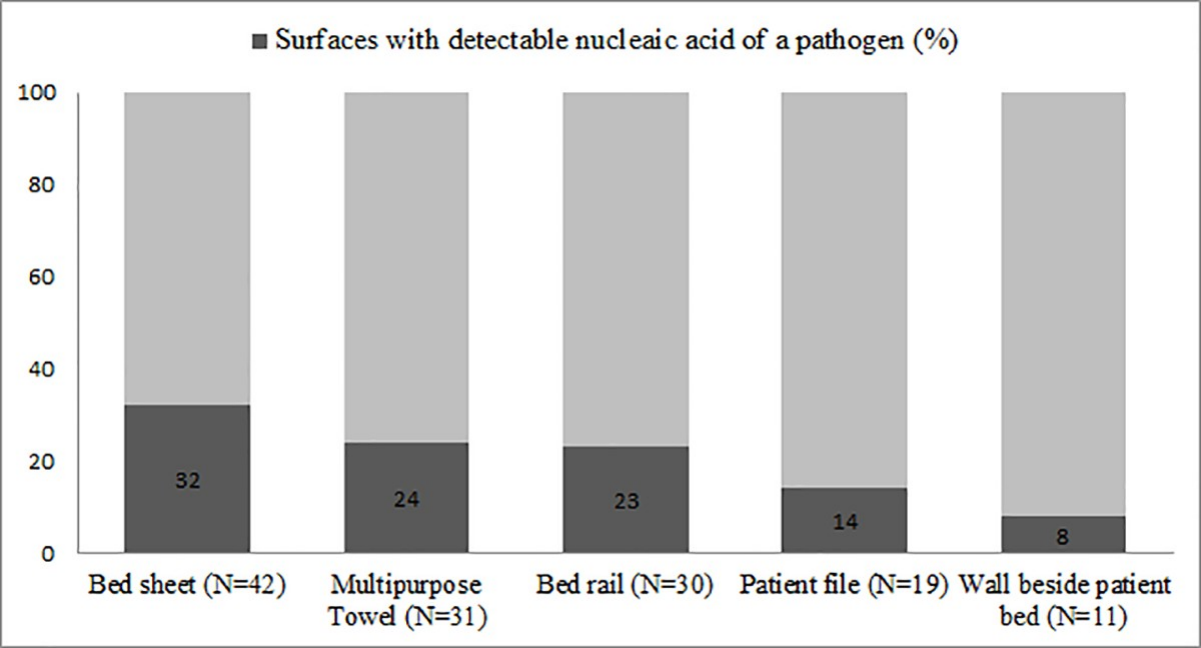
Percentage of different hospital surfaces* tested with detectable nucleic acid for common respiratory pathogens in two tertiary care hospitals in Bangladesh, September-November, 2013. *Surfaces collected nearby patients with at least one detectable pathogen in respiratory swabs.

We tested the surrounding hospital surface swabs for each of the 79 patients with evidence of respiratory pathogens for the same viral/bacterial nucleic acid detected in their respiratory swabs. We collected and tested 287 surface swabs near the 79 patients as not all these patients had a wall or bedrail near them. Nearly half of the hospital surface swabs (46% [133/287]) had evidence of contamination by at least one pathogen included in the testing panel. The most commonly contaminated surfaces were the bed sheet, the multipurpose towel, and the bed rail. We infrequently detected bacterial or viral nucleic acid on wall surfaces or on patients’ clinical record files (Fig 1). Sixty-two percent of patients (49/79) had detectable viral and/or bacterial nucleic acid on at least one (range: 1–5) nearby surface, including 60% of adults (18/30) and 63% (31/49) of pediatric patients. The most common bacterial pathogen detected on surface swabs was *Klebsiella pneumoniae* and 97% (32/33) of patients positive for *Klebsiella pneumoniae* had at least one surface with detectable DNA. The most frequently detected viral pathogen on surfaces was human cytomegalovirus and 86% (18/21) of patients positive for human cytomegalovirus had detectable DNA on nearby surfaces (Table 2).

**Table 2:** Frequency of surface contamination with detectable nucleic acid for specific pathogen near patients with detectable nucleic acid in respiratory swab of the same pathogen, in two tertiary care hospitals in Bangladesh, September-November, 2013.

Name of pathogen	Number of patients with detectable nucleic acid in respiratory swab	Pathogen detected on a nearby surface, n (%)
*Klebsiella pneumonia*	33	32 (97)
*Streptococcus pneumonia*	23	14 (60)
*Staphylococcus aureus*	13	8 (61)
Human cytomegalovirus	21	18 (86)
Human rhinoviruses	14	10(71)
Respiratory syncytial viruses	18	9 (50)
Other bacteria^a^	11	5 (45)
Other viruses^b^	17	15 (88)

^a^other bacteria: *Haemophilus influenza* (including type B), *Pneumocystis jiroveci* (fungi), *Bordetella pertussis*

^b^other viruses: human metapneumoviruses A and B, human bocavirus, enterovirus/human parechoviruses, adenovirus and influenza viruses

We tested nearby surfaces for 22 patients (18 patients from adult wards and 4 from pediatric wards) without detectable viral/bacterial nucleic acid in their respiratory swabs. We tested these surfaces for six frequently identified pathogens in respiratory swabs of case-patients: *Klebsiella pneumoniae*, *Streptococcus pneumoniae*, *Staphylococcus aureus*, human cytomegalovirus, respiratory syncytial viruses and human rhinoviruses. *Klebsiella pneumoniae* was detected on at least one nearby surface in 95% (21/22) of these patients, *Staphylococcus aureus* in 18% (4/22), and *Streptococcus pneumoniae* in 14% (3/22) patients. Viruses, including, human cytomegalovirus (4/22), respiratory syncytial viruses A and B (1/22) and human rhinoviruses (0/22), were rarely detected nearby these patients.

## Discussion

Nearly two-thirds of the patients hospitalized with laboratory-confirmed acute respiratory infection had at least one nearby contaminated surface. *Klebsiella pneumoniae* was the most commonly detected pathogen in patients' respiratory swabs, and was detected in nearly every environmental swab testing, suggesting widespread hospital contamination from current and previously hospitalized patients. With the ability to spread rapidly in the hospital environment, *Klebsiella pneumoniae* has been linked to several nosocomial outbreaks [19, 20]. The most frequently contaminated surfaces were the bed sheet, towel, and bed rail, further highlighting the perils of no routine surface cleaning practices in these hospitals. Studies in tertiary care hospitals of Bangladesh have shown that one in 20 patients with a hospital stay greater than three days developed a hospital-acquired respiratory infection, and that only 21% of those infections had a viral aetiology, suggesting a large proportion of these infections might be bacterial [21, 22]. Since, future work should further investigate the role of *Klebsiella pneumoniae* may be an important nosocomial pathogen in these hospitals.

Several other studies have identified multidrug resistant *Klebsiella pneumoniae* in hospital environments and its association with severe infections, prolonged hospital stays and increased mortality rates, particularly in debilitated and immunocompromised patients [23, 24]. A 2004 study in an urban tertiary care hospital in Dhaka, Bangladesh, reported that 40% of the clinical specimens (sputum, pus, urine, throat, and vaginal swabs) collected from hospitalized patients were drug resistant [25]. Moreover, our study findings were consistent between the two hospitals despite being located in different geographical areas and may suggest surface contamination with *Klebsiella pneumoniae* as a wider public health problem. The predominant bacterial pathogens we identified, *Klebsiella pneumoniae*, *Streptococcus pneumoniae*, and *Staphylococcus aureus*, can survive on surfaces from a few days to a few months [5, 26, 27]. Bacteria, in the presence of low humidity, forms biofilms protecting microorganisms from harsh environmental influences and are difficult to eradicate [28, 29]. In Bangladesh, hospital surfaces are not adequately cleaned and hospitals report insufficient supplies of cleaning agents [7, 8, 30]. To remove and prevent biofilm formation, hospital decontamination protocols should include strategies such as daily cleaning of surfaces with disinfectant (e.g. 0.3% sodium hypochlorite or 2% chlorhexidine solutions).

Among the most commonly detected viral pathogens, RSV was prevalent in respiratory swab of paediatric patients and nearby surfaces. RSV has been a major nosocomial hazard on pediatric wards and has been linked with hospital outbreaks [31, 32]. With reported higher case fatality among patient with nosocomial RSV infections (OR 4.46, 95% CI 1.1–18), widespread surface contamination with RSV is concerning for low income hospitals (34, 35).

We identified, that towel, was frequently contaminated with respiratory secretions. [8]. Islam *et al*. reported that family caregivers frequently used a multipurpose towel for patient secretions and for their own use, without cleaning it in between these uses [8].This suggests that the towel may act as a potential vehicle for transmission of respiratory viral and bacterial pathogens from patient to caregiver [33]. Infection control could target care giving practices associated with the use of the towel and should test feasible low-cost interventions such as the supply of low cost disinfectant by hospitals that encourage caregivers to clean the towels more frequently and to improve hand washing practices, including the use of hand sanitizer.

An important limitation of our study is that we only identified the presence of viral or bacterial nucleic acid on different hospital surfaces, and cannot be sure that the pathogens we detected were viable. A second limitation is that we conducted the study in only two public hospitals, so the findings may not be representative of all public hospitals. However, our findings were consistent between the two typical tertiary care hospitals we studied, located in completely different parts of the country. A third limitation is that we only sampled surfaces once, which limits our ability to comment on duration of contamination, and did not have the ability to observe contamination from seasonal infections. Influenza, for example, has a known seasonal pattern in Bangladesh, circulating usually between May and September, potentially explaining the low detection rate in our study [34, 35]. Lastly, we did not investigate drug resistance patterns of the bacteria we detected due to resource constraints. Based on evidence from other studies, however, it is likely that many of the pathogens we detected on surfaces were drug resistant and future studies should consider including investigations about drug resistance [25].

## Conclusions

This study identified that hospital surfaces in these Bangladeshi hospitals, were frequently contaminated with respiratory pathogens and pose a potential threat for fomite-borne transmission of respiratory infections to patients, healthcare workers and family caregivers. To prevent the spread of *Klebsiella* and other infections between patients, healthcare personnel must follow specific infection control precautions including strict adherence to hand hygiene and the use of gloves. In addition, our data clearly indicate that efforts to regularly disinfect environmental surfaces and ensure clean towels for patient caregiving could reduce risk of exposure to patients, healthcare staff and visitors. The government of Bangladesh has taken a number of initiatives to improve infection control in hospitals [36]. Despite this, a 2013 nationally representative survey of healthcare facilities showed that healthcare workers performed recommended hand hygiene in only 9% of 919 opportunities, suggesting low adherence to international standards [30]. Barriers include awareness, training, accountability and appropriate infrastructure (among 875 health facilities, 10% handwashing locations had no water, 20% had no soap and 50–80% had no alcohol based sanitizer) to support these behaviours [30, 37, 38] This study highlighted the gaps in practice, as well as the substantial barriers to improvement that will require widespread investments to address. In 2018, the Directorate of Hospital Infection Control, Directorate General of Health Services (DGHS) at the Ministry of Health and Family Welfare in Bangladesh has communicated the intention to form infection control committees in each district and tertiary care hospital across the country to improve the safe care [39]. Further investigation to identify the true contribution of fomites in the transmission of respiratory pathogens within hospital settings could be useful to help these committees prioritize efforts to improve hand and surface cleaning.

## Supporting information

S1 File Dataset. (DTA)
